# Event-related potentials indicate differential neural reactivity to species and valence information in vocal stimuli in sleeping dogs

**DOI:** 10.1038/s41598-023-40851-w

**Published:** 2023-09-04

**Authors:** Huba Eleőd, Márta Gácsi, Nóra Bunford, Anna Kis

**Affiliations:** 1 Department of Ethology, ELTE Eötvös Loránd University, Budapest, Hungary; 2 Doctoral School of Biology, Institute of Biology, ELTE Eötvös Loránd University, Budapest, Hungary; 3 ELKH-ELTE Comparative Ethology Research Group, Budapest, Hungary; 4 Research Centre for Natural Sciences, Institute of Cognitive Neuroscience and Psychology, Clinical and Developmental Neuropsychology Research Group, Budapest, Hungary; 5 Institute of Cognitive Neuroscience and Psychology, Research Centre for Natural Sciences, Budapest, Hungary, Budapest, Hungary; 6 ELTE-ELKH NAP Comparative Ethology Research group, Budapest, Hungary

**Keywords:** Perception, Sensory processing

## Abstract

Dogs live in a complex social environment where they regularly interact with conspecific and heterospecific partners. Awake dogs are able to process a variety of information based on vocalisations emitted by dogs and humans. Whether dogs are also able to process such information while asleep, is unknown. In the current explorative study, we investigated in *N* = 13 family dogs, neural response to conspecific and human emotional vocalisations. Data were recorded while dogs were asleep, using a fully non-invasive event-related potential (ERP) paradigm. A species (between 250–450 and 600–800 ms after stimulus onset) and a species by valence interaction (between 550 to 650 ms after stimulus onset) effect was observed during drowsiness. A valence (750–850 ms after stimulus onset) and a species x valence interaction (between 200 to 300 ms and 450 to 650 ms after stimulus onset) effect was also observed during non-REM specific at the Cz electrode. Although further research is needed, these results not only suggest that dogs neurally differentiate between differently valenced con- and heterospecific vocalisations, but they also provide the first evidence of complex vocal processing during sleep in dogs. Assessment and detection of ERPs during sleep in dogs appear feasible.

## Introduction

Being able to recognize and process incoming signals from social partners is necessary for successful participation in a social environment. One example are vocalisations emitted by conspecifics or heterospecifics that convey information ranging from information on supraindividual characteristics such as group or species membership to information on individual features such as age, sex, or emotional/motivational state^1,2^. Species membership and inner state of the caller are well studied characteristics (for a review see Ref.^2^ and also e.g., Ref.^3^).

Mammals spend a considerable proportion of their lifetime sleeping^4^. Being able to process environmental stimuli not only while awake but also while asleep is vital for survival. Temporal aspects of neural processing of environmental stimuli can be assessed using event-related potentials which, in humans, can be evoked during Stage 1 and Stage 2 non-REM sleep^5^. Extending on the literature with awake dogs^3^, here, we examine how sleeping dogs process differently valenced dog and human vocalisations at the neural level.

Dogs were integrated into the human environment over the years of domestication, and most communicate with both conspecific and heterospecific (human) partners on a regular basis. Behavioural studies show that dogs can match pictures of dogs^6^ and humans^7^ with their corresponding vocalisations, as well as dog and human emotional vocalisations with the congruent facial expressions^8^. Dogs also exhibit different behavioural responses to differently valenced vocalisations^9–11^.

These behavioural findings prompted questions about the neurophysiological correlates of emotional processing in dogs. An emerging number of studies are aimed at addressing related questions^12–14^ and the findings of these studies have largely contributed to the dog being considered a good model for comparative neurobiology^15^. Data indicate conspecific-sensitive and valence-sensitive brain regions in dogs and humans^16,17^. During electroencephalography (EEG), event-related potentials (ERPs) can be recorded and used to identify, with high temporal resolution, the effects of certain stimuli at the neural level^18^. ERPs in dogs have mainly been obtained using semi-invasive needle electrodes^19^ over the decades and more recently, a non-invasive method^20^. The latter method is completely painless, contrary to many other paradigms used in dog EEG studies (for a review of canine neurosurgery over history, see Ref.^21^), only surface electrodes are used, and participation is motivated solely by positive reinforcement (praise, treats).

Using the same non-invasive method, Bálint and colleagues examined the neural correlates of processing differently valenced dog and human vocalisations^3^. To test whether a species- or valence-specific difference of acoustic processing is observable, dogs were presented with dog and human vocalisations previously classified by human raters as either neutral or positive^56^. ERPs were recorded at two electrode sites, at the frontal and central positions of the anteroposterior midline of the skull (Fz, Cz). Findings showed human voices elicited a more positive ERP response than dog sounds in a time window ranging from 250 to 650 ms after stimulus onset. A species by valence interaction was also detected, such that in case of dog sounds, ERPs were more positive to neutral stimuli, whereas in case of human voices, ERPs were more positive to positive stimuli in a later time-window, ranging from 800 to 900 ms following stimulus onset. These results indicate that the dog brain differentially responds to the investigated factors. The species effect time-window coincides with two ERP components described in humans: the P300 and the earlier window of the late positive potential (LPP), both of which are generally associated with emotional processing and attention to motivationally significant stimuli^22–25^. The species by valence interaction time-window can be interpreted as indexing more subtle processing of the vocalisations, attributed to higher-level cognitive processes^26^.

Dog and human sleep show several parallels: e.g., regarding sleep-dependent memory consolidation^27,28^ and processing of pre-sleep emotional treatment^29^. It has even been proposed that relative to commonly used laboratory animals, the general architecture of human sleep is better approximated by dog sleep^30^. It remains unknown however, whether similarly to humans, processing of auditory signals can be indexed by ERPs during sleep in dogs (for a review of human ERP studies during sleep, see Ref.^31^).

The ability to efficiently process the conspecificity or the emotional state of the caller has been demonstrated in behavioural studies in primates^32,33^ and non-primates^34,35^. The emotional content of vocalisations is also important in humans, and it has been shown to modulate neural processing even in neonates (i.e., infants younger than 28 days) and infants during active sleep^36^. Although in most studies with humans, focus was on visual cues, neural processing was affected by emotional speech (for a review, see Ref.^37^) and by nonverbal emotional vocalisations^38,39^. Data further show that humans are able to process emotional stimuli while asleep and that certain brain regions implicated in emotion processing are more active during sleep than in when awake^40^ and that ERPs of acoustic processing, such as K-complexes^41^ and other long latency components^42^ have been observed even during deep-sleep (SWS).

In the present study, we investigated whether dog brains differentially respond to species and valence information in vocal stimuli during different sleep stages. We expected to find, as in awake dogs^3^, species- and valence-sensitivity, especially during light sleep (drowsiness).

## Results

### Descriptive results

**Figure 1:**
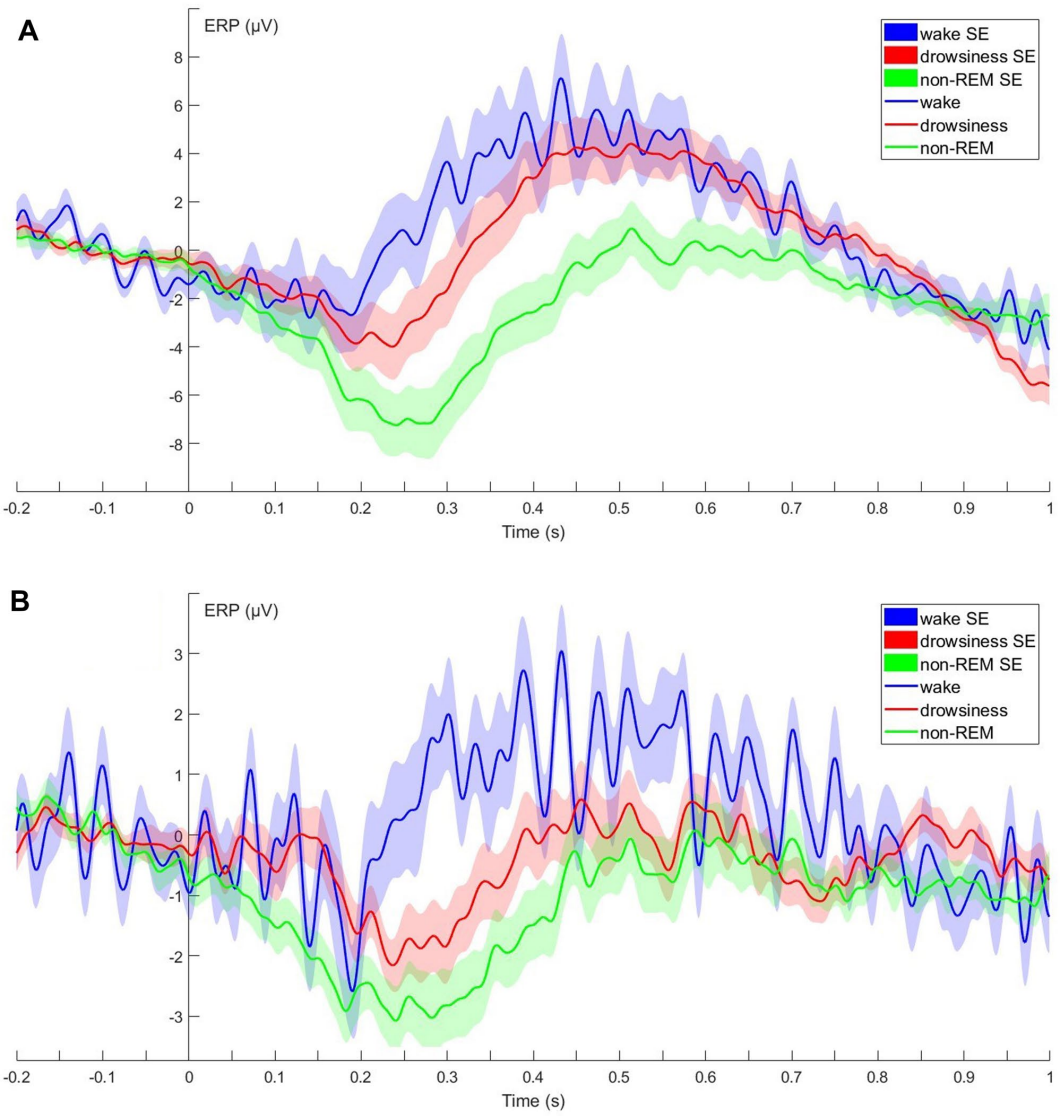
ERPs recorded at Fz (**A**) and Cz (**B**) during wake (blue), drowsiness (red) and non-REM (green) from 200 ms before to 1000 ms after stimulus onset (0 point on the x-axis).

Grand average ERP waveforms during awake, drowsiness and non-REM stages (irrespective of stimulus type) are depicted in Fig. 1. Visual inspection of the data suggests a difference in the temporal onset of the evoked response (on both the Fz and Cz electrode) with earlier ERP onset while awake, followed by drowsiness and then by non-REM. The averages also suggest more positive ERPs from approximately 200 to 700 ms in wake, followed by drowsiness and then by non-REM. However, as subjects included in analyses of different sleep stages overlap (thus neither truly between-subject, nor truly within-subject) no statistical analysis was performed to confirm the observed trends.

### Sliding window analysis

To assess whether characteristics of the vocal cues affected ERPs, we conducted a sliding-window analysis on 1-s-long segments averaged for each dog. The interval from 0 to 1000 ms (0–1000 ms) was analysed with 100 ms long overlapping windows (between 0 and 100 ms, 50 and 150 ms, 100 and 200 ms etc.). When consecutive 100 ms time windows showed a statistically significant ERP modulation effect, these were further averaged and analysed as a single, conjoined window.

Results are reported below as statistically significant if *p* < 0.05 (also presented in Table 1. in detail). We also report the direction of non-significant trends (*p* < 0.1) to indirectly compare the direction of the associations obtained in previous studies^3^.

**Table 1:** Results of the statistical analysis.

Sleep-stage	Electrode	Time-window (ms)	Factor	NumDF	DenDF	F value	Pr (> F)
Wake	Fz + Cz	750–850	Valence:species	1	31	3.665	0.065
		400–600	Electrode	1	31	5.043	0.032
		850–1000	Electrode	1	31	6.706	0.015
	Cz	650–750	Species	1	12	3.95	0.070
Drowsiness	Fz + Cz	250–450	Species	1	66	5.839	0.018
		600–800	Species	1	63	6.579	0.013
		50–200	Electrode	1	54	4.417	0.040
		350–800	Electrode	1	66	10.122	0.002
		800–1000	Electrode	1	66	46.226	< 0.005
	Cz	250–450	Species	1	27	8.222	0.008
		600–800	Species	1	27	7.175	0.012
		250–400	Valence:species	1	27	5.293	0.030
Non-REM	Fz + Cz	200–350	Species	1	38	3.398	0.073
		100–350	Electrode	1	38	8.976	0.005
		700–1000	Electrode	1	38	11.454	0.002
	Cz	750–850	Valence	1	11	5.233	0.043
		200–300	Valence:species	1	10	6.867	0.026
		450–650	Valence:species	1	10	6.545	0.028

### Wake

With both electrodes in the model, there was a trend-level species × valence interaction effect from 750 to 850 ms (LMM: F_1,17_ = 3.67; *p* = 0.064). The positive dog and the neutral human vocalisations (classification made by human raters^56^) corresponded to a more positive ERP effect.

Electrode site had a main effect, with more positive ERPs on Fz compared to Cz between 400 and 600 ms after stimulus onset (incorporating 3 consecutive time-windows, LMM: F_1,31_ = 5.04; *p* = 0.032). Electrode site also had a main effect, with more positive ERPs on Cz from 850 to 1000 ms (2 consecutive time-windows, LMM: F_1,17_ = 6.71; *p* = 0.015).

Analysing the two electrodes separately, we registered no effect on Fz.

On Cz, there was a trend-level main effect of species from 650 to 750 ms (LMM: F_1,12_ = 3.95; *p* = 0.070), with more positive ERPs after dog than human voices.

### Drowsiness

**Figure 2:**
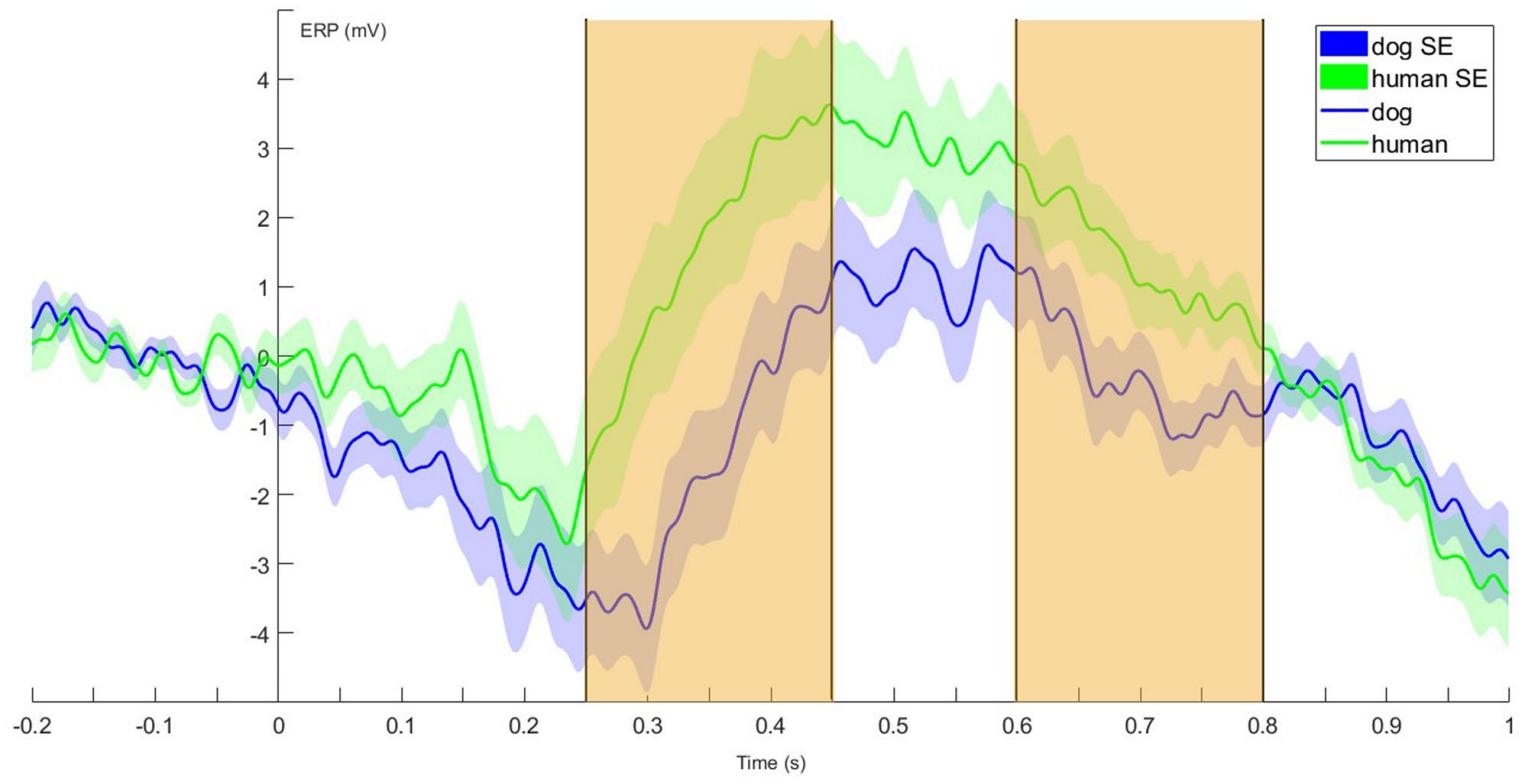
Grand-averaged ERPs showing the average of the two levels of the species factor, from 200 ms before to 1000 ms after stimulus onset (0 point on the x-axis). The highlighted parts designate the time windows between 250 and 450 ms and between 600 and 800 ms where the species effect was significant in the sliding time-window analysis during drowsiness.

With both electrodes in the model, there was a main effect of species from 250 to 450 ms after stimulus onset (incorporating 3 consecutive time-windows) (LMM: F_1,66_ = 5.839; *p* = 0.018) and from 600 to 800 ms (incorporating 3 consecutive time-windows) (LMM: F_1,64_ = 6.579; *p* = 0.013). In both cases, ERPs were more positive to human than to dog stimuli (Fig. 2).

There was a species × valence interaction effect from 550 to 650 ms (LMM: F_1,65_ = 4.267; *p* = 0.043). ERPs to positive and neutral stimuli differed depending on the species of the caller: responses were more positive to positive stimuli in case of dog vocalisations, whereas they were more positive to neutral stimuli in case of human acoustic cues.

Electrode site had a main effect in three combined time-windows: more positive Cz responses were apparent from 50 to 200 ms (2 consecutive time windows; LMM: F_1,54_ = 4.417; *p* = 0.040), more positive Fz responses from 350 to 800 ms (8 consecutive time windows; LMM: F_1,66_ = 10.122; *p* = 0.002), and more positive Cz responses were detected from 800 to 1000 ms (3 consecutive time windows; LMM: F_1,66_ = 46.226; *p* < 0.001).

Analysing the two electrodes separately, we registered no effect on Fz.

Analysis of Cz showed a main effect of species from 250 to 450 ms, incorporating three consecutive time-windows (LMM: F_1,27_ = 8.222; *p* = 0.008) and from 600 to 800 ms, incorporating three consecutive time-windows (LMM: F_1,27_ = 7.175; *p* = 0.012). In both cases, ERPs were more positive to human relative to dog vocalisations.

There was a species × valence interaction from 250 to 400 ms, incorporating two consecutive time-windows (LMM: F_1,27_ = 9.585; *p* = 0.004) such that in case of dog sounds, more positive cues elicited more positive ERPs, contrary to human vocalisations, where neutral cues produced such effect.

### Non-REM

Analysing the two electrodes together, there was a trend-level main effect of species (2 consecutive time-windows between 200 and 350 ms; LMM: F_1,38_ = 3.40; *p* = 0.073). Dog sounds tended to elicit a more positive ERP response than human sounds.

Electrode site had a main effect in two separate time-windows: more positive Cz responses were found from 100 to 350 ms (4 consecutive time-windows; LMM: F_1,38_ = 8.98; *p* = 0.005) and also from 750 to 1000 ms (4 consecutive time-windows; LMM: F_1,38_ = 11.45; *p* = 0.002).

Analysing the two electrodes separately, we registered no effect on Fz.

**Figure 3:**
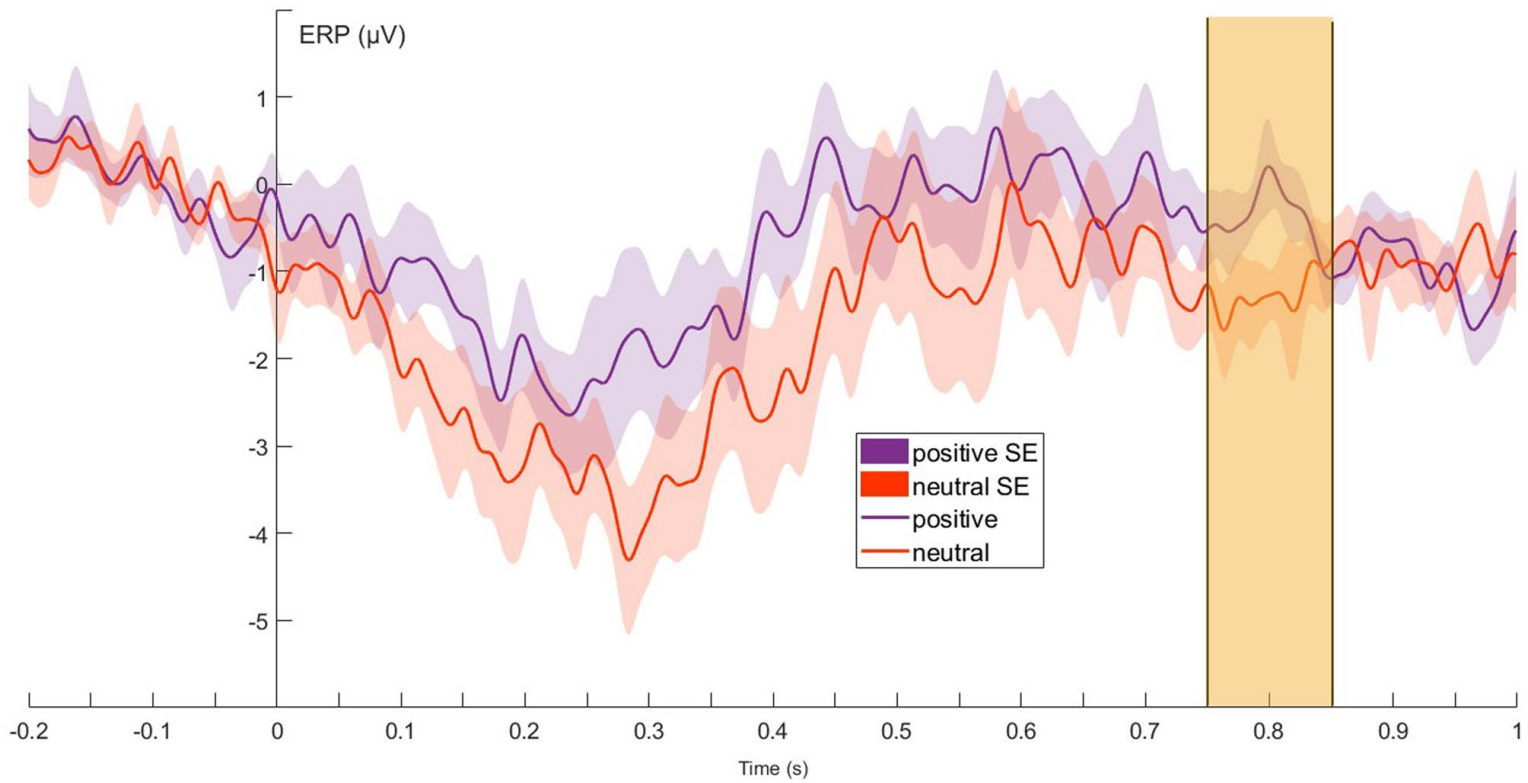
Grand-averaged ERPs showing the average of the two levels of the valence factor, from 200 ms before to 1000 ms after stimulus onset (0 point on the x-axis). The highlighted part designates the time window between 750 and 850 ms where the valence effect was significant in the sliding time-window analysis on Cz during non-REM.

On Cz, there was a main effect of valence (Fig. 3.) from 750 to 850 ms (LMM: F_1,11_ = 5.233;* p* = 0.043), where ERPs were more positive after positively valenced compared to neutral vocalisations.

There was a species × valence interaction effect between 200 and 300 ms (LMM: F_1,10_ = 6.867; *p* = 0.026) and from 450 to 650 ms (3 consecutive time-windows; LMM: F_1,10_ = 6.545; *p* = 0.028), with more positive ERPs after positive stimuli in case of dog voices and neutral cues in case of human voices.

## Discussion

Here we provide the first evidence that in addition to the awake state, event related potentials can be evoked in dogs during (drowsiness and non-REM) sleep. Using differentially valenced acoustic stimuli from dogs and humans (the valence rating conducted by human listeners in a previous study^56^), we conclude that the dog brain can differentiate between vocalisations based on species and valence factors in two sleep-stages, similarly to how the human brain is able to process various characteristics of acoustic cues during sleep^31^. This finding is significant insofar as it is the first evidence of complex auditory processing during sleep in dogs.

It can be easily detected that behaviorally, awake dogs differentially respond to vocal cues depending on different characteristics of these sounds. For example, dogs differently react to the vocal cues of conspecifics, based on familiarity^43^ or body size^44^; and they also differentiate between human vocal cues based on emotions^8^ or caller identity^7^. Such reactions are relatively straightforward to quantify in the form of e.g., looking durations or other behavioural responses. Thus, although research on awake dog fMRI^16^ and ERP^3,19,20,45^ measures of auditory processing has expanded the available behavioural literature (and provided insight into biological mechanisms), the assessment of neural response is essential for examining whether dogs are able to detect and process vocal stimuli during sleep and what characteristics of these acoustic cues are they reactive to, in the absence of behaviour that would be observable.

In the current exploratory study, we found that dog brains process species (human versus conspecific) as well as valence (rated as positive versus neutral) information during deep / non-REM sleep. Although the sample size was small and direct comparisons between different sleep stages was not feasible given the distribution of artefacts, these results indicate that the non-invasive ERP protocol we applied is a feasible method to examine vocal stimuli processing in sleeping dogs.

Our results are also consistent, in part, with awake dog ERP data involving the same pool of stimuli, in that effects were found in comparable time windows, though in certain cases, in a different direction^3^. In some cases, for example the herein observed effects are only partially overlapping with those previously reported: during drowsiness, a species main effect was observed in two time windows ranging from 250 to 450 ms and from 600 to 800 ms, whereas in awake dogs this effect lasted from 250 to 650 ms (further discussed below). It should be noted that a relevant consideration in interpreting the current findings and in comparing those to data obtained previously is that in Bálint et al., dogs were trained to lie motionless. We have no data on how such training (and its results, e.g., associations between the test situation and rewards) might have affected emotion processing. Of note however, as dogs participated in several dozens of assessment sessions in Bálint et al., they all reached a calm, relaxed state during that experiment. Given the length of the sessions and that dogs only received a food reward at the end of each session in this previous study, it is likely that there were no reward anticipation effects.

In some cases, the direction of the current results was opposite to those described earlier^3^. In the current sample, a non-significant interaction of main factors emerged in the wake stage between 750 to 850 ms: ERPs were more positive after positive cues coming from a dog (compared to to neutral dog sounds), whereas ERPs were more positive after neutral cues coming from humans (relative to positive human sounds). The opposite was the case in Bálint et al., where neutral dog sounds and positive human sounds elicited more positive ERPs in a time window ranging from 800 to 900 ms. The current dataset contained only a limited amount of wake data, and as the observed effect did not reach statistical significance, it might easily reflect a non-effect. However, mainly on Cz, the same association (more positive ERPs to positive dog and to neutral human stimuli) was significant during drowsiness and non-REM sleep. On the other hand, dogs participating in the current study were untrained and they were more relaxed, dozing off even during periods categorised as wake. Thus, differential responses to the same emotionally valenced dog and human vocalisations might be related to the state that the subject is in: different stimuli are relevant to a more alert individual participating in a task compared to a relaxed individual about to fall asleep. We must also note that Bálint et al. described a large individual variation in responses to the vocalisations represented by the four conditions (for individual graphs, see Fig. S1 in Ref.^3^). Since the sample size in the current study was rather small, it cannot be excluded that some group effects are due to the composition of the sample.

We detected a species effect in two time windows ranging from 250 to 450 ms and from 600 to 800 ms in drowsiness. In dogs, drowsiness has been described as a transition stage between being awake and asleep^46,47^, so comparison of findings with those described in studies of awake animals and of those in superficial (S1) sleep can both be justified. Our findings overlap with the previously described window lasting from 250 to 650 ms^3^ in awake dogs, indicating that the same neural mechanisms may be taking place during this transitional stage. The later time-window is conceptualised as the late positive potential (LPP) ERP component in humans, a sustained positivity beyond the P300 that reflects elaborate processing of and sustained attention to affectively and motivationally salient stimuli^23–25,48^. These findings also share further characteristics with phenomena reported in awake dogs^3^, as in both cases the human voices produced more positive ERP waves with bigger amplitudes than their dog vocalisations.

The valence-species interaction found between 550 and 650 ms may also reflect data on the LPP in humans indicating an ‘Emotion’ by ‘Voiceness’ interaction during 500–800 ms^49^ that has been interpreted as reflecting independent neural processing of these two factors before being integrated at a later and more controlled processing stage.

During non-REM sleep (SWS), the effect of valence registered on Cz in a time-window from 750 to 850 ms may also reflect this later, more refined processing of acoustic cues. ERPs have been shown to be present even during stage 2 and slow wave sleep (SWS) in humans^50^, as certain relevant stimuli (e.g., the participant’s name) are processed even during stage 2 and paradoxical (REM) sleep^51^.

In combination with the fMRI study of Andics et al.^16^, the current results can also provide insight into the underlying brain mechanisms. Using partly the same vocal cues, Andics et al. registered species- and valence-sensitive areas in the dog brain. The ERP method is generally not considered as spatially accurate, thus direct comparison of these results with fMRI data is not possible, but the difference in processing of these sounds based on the main factors further support the divergent underlying processing of these two types of vocalisations.

**Figure 4:**
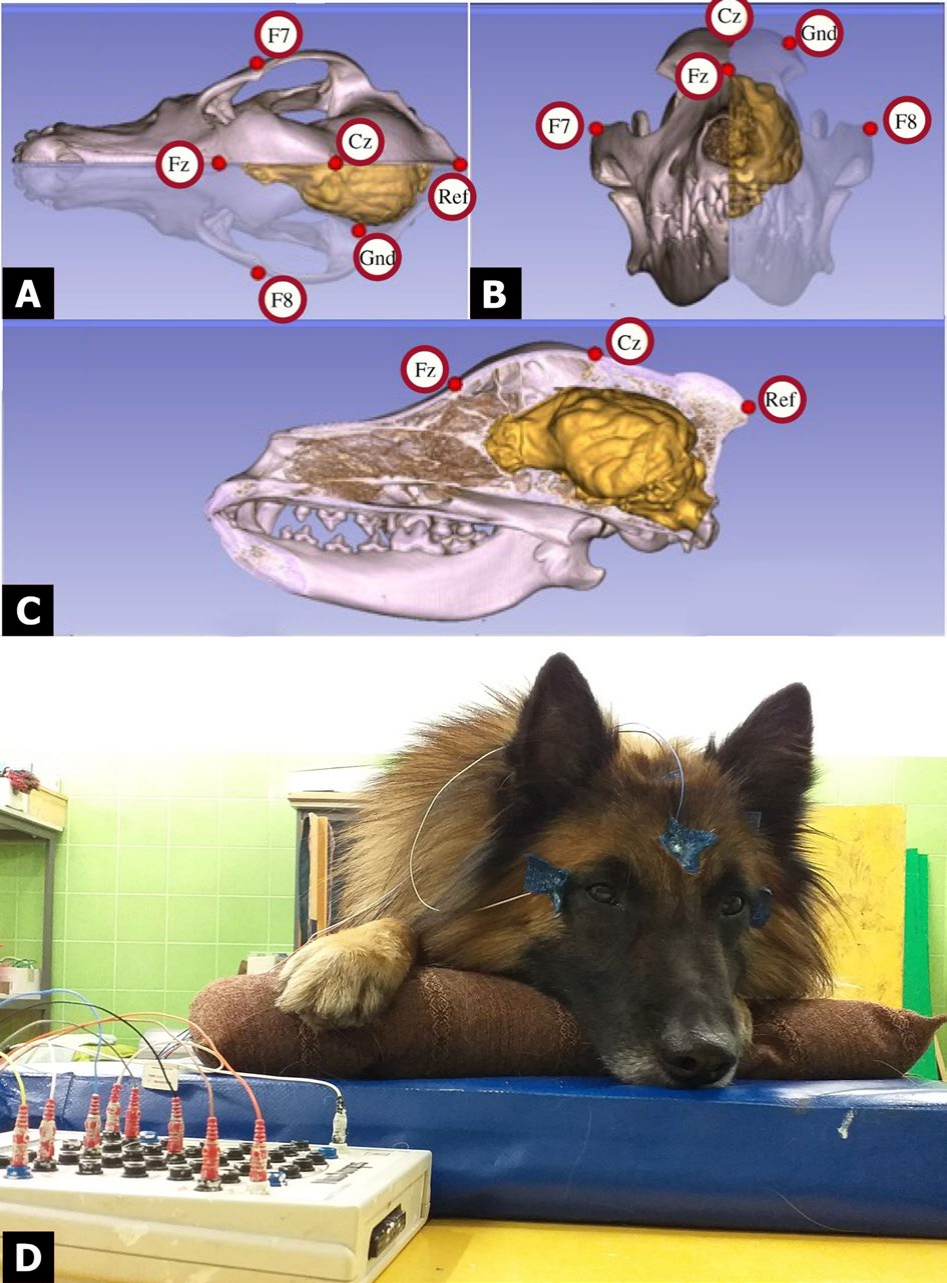
Positions of the Fz and Cz electrodes relative to the three-dimensional model and endocranial cast of the skull of a pointer dog (with yellow showing the brain's morphology), image modified from Fig. 1 in Ref.^3^, (**A**) superior (**B**) anterior and (**C**) lateral views, images courtesy of Kálmán Czeibert (**D**) Photograph showing a dog during the measurement, photo by Huba Eleőd. A video showing the electrode placement procedure can be found at: https://youtu.be/OYc7ALKtowk.

The electrode effects found during all three sleep-stages were expected based on previous findings^3^, where this effect was reported to last from 350 to 700 ms. The electrode effect probably reflects differences in the distribution of the electrodes on the scalp: as Cz was closer to the reference, it generally registered smaller ERP amplitudes. Three other factors, electrode distance from the brain; electrode distance from certain acoustically sensitive brain areas, or thickness of the ventriculus (the bone and other tissues between the recording sites and the brain; illustrated in Fig. 4) would also result in these differences. These data are consistent with fMRI findings (e.g., Ref.^16^), where regions sensitive to emotional valence and species have been located relatively closer to where the Cz electrode was placed. The lack of any effects on Fz indicates that there is a need for reconsideration, and further calibration, of electrode placement in dog ERP studies, even including electrode placement on the side of the skull that would permit assessment of laterality (something that the current design was not appropriate for).

The current study is not without limitations. As most limitations are due to methodological difficulties, the feasibility of the sleep ERP paradigm is important to discuss. Although *N* = 13 dogs were enrolled, analyses could be carried out on only *N* = 10 of these for drowsiness, and on *N* = 6 and *N* = 5 for wake and non-REM respectively. Also, none of the recordings contained enough artefact-free REM traces (more than 10 artefact-free trials in all four conditions). While dogs are awake, they are expected to produce muscle tone artefacts (see e.g., Ref.^46^), thus we were not surprised to see that, compared to our previous (well-trained to stay motionless) sample, wake data were not so clear here. REM sleep has also been omitted from some of the previous studies (e.g., Ref.^52^) due to eye-movement artefacts inherent to this stage; although it would be very interesting to include REM in an ERP study and examine how dog brains process vocal stimuli in this sleep stage. It was surprising to see, however, that only half of the dogs produced meaningful non-REM data, as this is usually the stage with the clearest EEG traces^53^. One explanation for this is that the stimuli presented in the current study also served as sleep disturbance and produced micro-arousals or even awakenings. Based on these experiences we conclude that although use of a non-invasive ERP paradigm with dogs is quite labour-intensive, it is nevertheless the most feasible possibility to study vocal stimuli processing during sleep, as it does not require prior training and does not cause any distress or harm.

In summary, we show for the first time that dogs also process information about emotional valence and species during sleep, actively reacting to the stimuli of their surroundings even in deep-sleep stages. These findings, parallel to those described previously^3^ improve our understanding of the dog brain and offer both a new tool for canine acoustic ERP studies and a new subject species for comparative neuroimaging experiments.

## Methods

### Ethical statement

Owners were recruited from the Family Dog Project (Eötvös Loránd University, Department of Ethology) database and participated in the study following written informed consent and without monetary compensation. The research was carried out in accordance with the Hungarian regulations on animal experimentation and the Guidelines for the Use of Animals in Research described by the Association for the Study Animal Behaviour (ASAB). All experimental protocols were approved by the Scientific Ethics Committee for Animal Experimentation of Budapest, Hungary (No. of approval: PE/EA/853-2/2016).

The design of the study and the methods used are reported in accordance with ARRIVE guidelines (https://arriveguidelines.org/arrive-guidelines).

### Subjects

*N* = 13 family dogs participated, 9 females and 4 males, age: 1–10 years (mean ± SD = 5 ± 2.9 years), five mixed breeds, two golden retrievers, one English cocker spaniel, one German pinscher, one small Münsterlander and one Airedale terrier.

### Electrophysiological recordings

EEG recordings were carried out during the dogs’ daily afternoon nap, following guidelines originally described in Ref.^46^ and implemented across several studies since (e.g., Refs.^3,29,54,55^). Briefly, the method involves parallel recording of several physiological parameters with electroencephalogram (EEG), electrooculogram (EOG), electrocardiogram (ECG) and a respiratory belt. In the current study, only the EEG data were used for analyses, the other physiological parameters were only used to aid sleep stage detection.

We applied surface attached, gold-coated Ag/AgCl electrodes onto the surface of the skin, secured by EC2 Grass Electrode Cream (Grass Technologies, USA). The electrode sites we used were on the frontal and central positions of the anteroposterior midline of the skull (Fz, Cz), both referenced to an electrode at the posterior midline of the skull (Ref; occiput/external occipital protuberance). Two electrodes on the right and left zygomatic arch, next to the eyes, served to detect eye movements (EOG: F7, F8). The ground electrode (Gnd) was placed on the left musculus temporalis (Fig. 4). Impedance values were kept below 20 kΩ.

The signals were amplified by a 40-channel NuAmps amplifier (© 2018 Compumedics Neuroscan) and digitised at a sampling rate of 1000 Hz/channel, applying DC-recording.

### Experimental set-up

**Figure 5:**
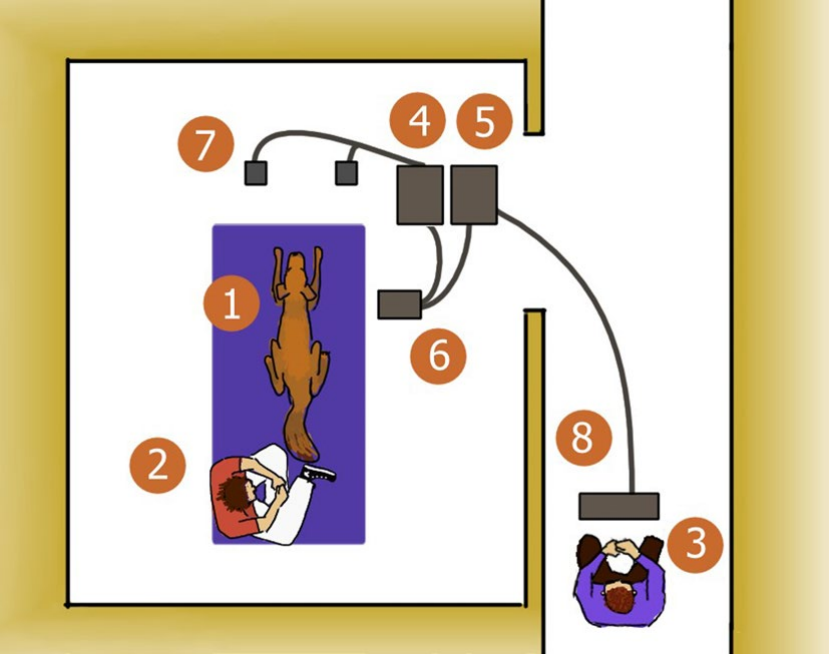
The general layout of the lab. (1) Dog subject; (2) owner; (3) experimenter; (4) computer: presenting the stimuli; (5) computer: recording the signal; (6) EEG amplifier; (7) speakers; (8) monitor.

The experiments were conducted in a 5 × 6 m laboratory equipped for neurophysiological measurements at the Department of Ethology, University of ELTE (for the general layout of the lab see Fig. 5). Upon arrival, the experimenter informed the owner about the conditions of the experiment, while the dog was left to freely explore the room (5–10 min). Then the owner and the dog were asked to take place on a mattress which was placed right next to the recording and trigger distributing computers. Acoustic stimuli were presented from two speakers (Logitech X-120) placed 1 m away from the mattress and 1 m apart from each other, to ensure an even distribution and hearability of the stimuli. An additional PC screen was connected to the recording computer to assist the experimenter in monitoring the recording during sleep. When the dog settled, the experimenter started the electrode attachment process, while constantly monitoring the signal of the recording electrodes, verifying that their impedance values were below 20 kΩ. When all signals were satisfactory and the dog assumed a lying, relaxed position next to its owner, the experimenter started the recording and the stimulus presentation program, turned off all lights (except for a reading lamp, if requested by the owner) and left the room. The experimenter then assumed a position outside of the room and monitored the recording. During the experiment, owners were asked to read or sleep without using their mobile phones.

### Stimulus presentation

The acoustic stimuli were identical to those described in a recent article^3^ and consisted of non-verbal vocalisations collected from dogs and humans, rated by human listeners in a previous study on 100 point scales of the perceived valence rating and intensity^56^. Altogether 10 positive and 10 neutral dog vocalisations as well as 10 positive and 10 neutral human (non-verbal) vocalisations were used, the same as earlier^3^. These sounds had received either the highest emotional valence scores (positive), or the lowest absolute value scores (neutral) previously^51^ and were from different call types (positive dog: grunt, pant, growl, moan and whine; neutral dog: yelp, bark, moan and grunt; positive human: general and laugh; neutral human: moan, sigh, yawn, general and cough. During the selection process sounds with distinctive sexual undertones (sexual moans) were omitted. Details of the stimuli are presented in the Supplementary material.

Only positive and neutral cues were used to achieve multiple goals, the first one being the issue of comparability: by using the same set of cues, our results can be directly compared to the ones found in a previous experiment^3^. In the present study we also wanted to avoid waking or startling the dogs by playing them extremely negatively valenced stimuli while they were asleep. All sound-files had the same duration (1 s), and the volume of the sound-files was the same across conditions (one-way ANOVA: M(all) = 69.75 dB, s.d. = 1.51, F_1_ = 0.16, *p* = 0.70). The signals followed each other (interstimulus interval was randomised between 19 and 25 s) in a manner that no three stimuli followed each other from the same condition. One session lasted approximately 3 h.

### Data analysis

Recordings were divided into 20 s long epochs, and each epoch was visually classified as awake, drowsiness, non-REM, or REM according to standard criteria using the Fercio program (see Ref.^53^ for the validation of the scoring system and data on interrater agreement). The program was developed and is being continuously updated since 2009 and has been employed in many studies (e.g., Refs.^46,53^). It allows for simultaneous examination of all relevant polysomnographic channels (in our case the EEG data derived from Fz and Cz electrodes, EOG data from F7 and F8 channels, respiratory and ECG data).

Sleep scoring was followed by the analysis of evoked potentials in an identical way as described in a former study^3^, but separately for the different sleep stages. EEG pre-processing and artefact rejection were conducted using the FieldTrip software package^57^ in Matlab 2014b. The continuous EEG recording was filtered using a 0.01 Hz high-pass and a 40 Hz low-pass filter, then 1200 ms long trials were segmented with a 200 ms long pre-stimulus and a 1000 ms long interval after the onset of the stimulus. These trials then were detrended (removing linear trends) and baselined (using the 200 ms long pre-stimulus interval). Then an automatic rejection process was carried out, removing all trials with amplitudes exceeding ± 150 µV and differences between minimum and maximum amplitude values exceeding 150 µV in 100 ms sliding windows (automatic rejection). This was followed by a visual inspection of each trial, where the remaining trials with muscle artefacts or missing signal on either of the analysed electrodes were removed. During this step the person conducting the analysis was blind to the condition of the stimuli.

Based on trial numbers used in infant studies (e.g., Refs.^58,59^) and a previous dog ERP study^3^, we excluded subjects from the analysis if less than 10 trials were left in any of the conditions after the artefact rejection process. However, if a subject was excluded from the analysis of one sleep stage (due to the below threshold number of artefact-free traces) it could still be included in the analysis of another sleep stage if it reached threshold there; in fact if a dog had the sufficient number of artefact-free traces during the wake stage, it typically meant that it spent less time sleeping during the recording and thus did not reach threshold for drowsiness and / or non-REM (and the other way round). Unfortunately, none of the dogs produced the sufficient number of artefact-free traces for the REM stage (mostly due to the rapid eye-movements inherent to REM, which result in EEG artefacts, especially in our case, when the presence of the vocal cues presumably resulted in a more superficial sleep), thus it could not be analysed. The final sample thus consisted of *N* = 5 subjects for analyses involving the wake (Subjects 4, 8, 10, 11, 13), *N* = 6 dogs for analyses involving the non-REM (Subjects 1, 2, 3, 5, 9, 13) and 4 additional, thus *N* = 10 dogs (Subjects 1, 2, 3, 4, 5, 7, 9, 11, 12, 13) for analyses involving the drowsiness sleep-stage.

Overall our final dataset contained 94 ± 32 trials per subject (positive-dog = 23 ± 5.8, neutral-dog = 23 ± 7.1, positive-human = 23 ± 9.3, neutral-human = 25 ± 11) in the wake stage; 67 ± 25.7 trials per subject (positive-dog = 17 ± 6.9, neutral-dog = 16 ± 7.6, positive-human = 19 ± 6.4, neutral-human = 16 ± 6.9) in the drowsiness stage and 115 ± 49.4 trials per subject (positive-dog = 27 ± 12, neutral-dog = 31 ± 14, positive-human = 30 ± 13, neutral-human = 28 ± 12) in the non-REM sleep-stage.

### Statistical analysis

We conducted a linear mixed model (LMM) analysis in R (R Core Team, 2018), with a model described in detail in a former article^3^. It was a result of a backward elimination process by comparing the Akaike information criterion score of potential models using a top-down approach, starting with the main factors (valence, species, electrode), their interactions and random slopes of the main factors. The final, best-fitting model consisted of the species, valence, and electrode site as main factors, a species by valence interaction, and an additional random slope of valence. The EEG traces were systematically analysed by performing a 100 ms consecutive time-window analysis on the segments averaged for each dog. The interval from 0 to 1000 ms (0–1000 ms) was analysed with 100 ms long overlapping windows (between 0 and 100 ms, 50 and 150 ms, 100 and 200 ms etc.). When consecutive 100 ms time windows showed a statistically significant ERP modulation effect, these were further averaged and analysed as a single, conjoined window.

### Supplementary Information

Supplementary Information.

## Data Availability

The datasets and scripts used in the study can be accessed via the following link: https://drive.google.com/drive/folders/1nN2Oznh3i4WGvLQpr4mygrqOd1omSx5H?usp=sharing.
