# Supplementary material for: Event-related potentials indicate differential neural reactivity to species and valence information in vocal stimuli in sleeping dogs
Source: Sci Rep. 2023 Sep 4;13:14518. doi: 10.1038/s41598-023-40851-w (PMC10477275; doi:10.1038/s41598-023-40851-w)
Supplement: Supplementary file 1 — Supplementary Information. [file 41598_2023_40851_MOESM1_ESM.docx]

| Positive-dog | | | Neutral-dog | | | Positive-human | | | Neutral-human | | |
| --- | --- | --- | --- | --- | --- | --- | --- | --- | --- | --- | --- |
| ID | Call type | Valence score | ID | Call type | Valence score | ID | Call type | Valence score | ID | Call type | Valence score |
| pdog1 | Grunt | 31,32 | ndog10 | Yelp | 4,47 | phum2 | Laugh | 40,82 | nhum4 | Moan | 3,11 |
| pdog2 | Grunt | 23,14 | ndog3 | Bark | 1,97 | phum6 | General | 37,36 | nhum5 | Sigh | 1,32 |
| pdog3 | Pant | 23,05 | ndog2 | Bark | 1,86 | phum1 | Laugh | 36,59 | nhum10 | Yawn | 0,31 |
| pdog6 | Growl | 22,56 | ndog1 | Bark | 0,97 | phum7 | Laugh | 36,33 | nhum2 | Yawn | 0,26 |
| pdog8 | Moan | 21,26 | ndog4 | Bark | 0,42 | phum8 | Laugh | 35,87 | nhum1 | General | -0,43 |
| pdog4 | Grunt | 20,64 | ndog6 | Moan | 0,34 | phum4 | Laugh | 35,59 | nhum8 | General | -1,24 |
| pdog7 | Moan | 16,00 | ndog8 | Bark | 0,30 | phum10 | Laugh | 34,46 | nhum3 | Sigh | -1,55 |
| pdog5 | Moan | 15,91 | ndog5 | Yelp | -0,03 | phum9 | General | 32,53 | nhum6 | Sigh | -3,39 |
| pdog10 | Moan | 12,15 | ndog9 | Grunt | -2,46 | phum5 | General | 30,39 | nhum9 | Cough | -9,86 |
| pdog9 | Whine | 11,54 | ndog7 | Moan | -2,92 | phum3 | General | 29,34 | nhum7 | Cough | -12,82 |

**Supplementary table S1.** List of stimuli presented for the human & dog positive & neutral conditions. The ID number was randomly assigned to sounds within each condition. Call types were categorized by an expert coder in Faragó et al. 2014^57^. Valence mean values are based on the rating of N=39 subjects (see^57^); scoring was done on a scale from -50 (negative) to 50 (positive), with values close to 0 being neutral.
